# Altered Hippocampal Subfields Volumes Is Associated With Memory Function in Type 2 Diabetes Mellitus

**DOI:** 10.3389/fneur.2021.756500

**Published:** 2021-11-25

**Authors:** Mingrui Li, Yifan Li, Yujie Liu, Haoming Huang, Xi Leng, Yuna Chen, Yue Feng, Xiaomeng Ma, Xin Tan, Yi Liang, Shijun Qiu

**Affiliations:** ^1^First Clinical Medical College, Guangzhou University of Chinese Medicine, Guangzhou, China; ^2^Department of Radiology, The First Affiliated Hospital of Guangzhou University of Chinese Medicine, Guangzhou, China

**Keywords:** type 2 diabetes mellitus, hippocampal subfields, cognitive function, volumetry, magnetic resonance imaging

## Abstract

**Objective:** Cognitive impairment in type 2 diabetes mellitus (T2DM) patients is related to changes in hippocampal structure and function. However, the alternation of hippocampal subfields volumes and their relationship with cognitive function are unclear. This study explored morphological alterations in the hippocampus and its subfields in T2DM patients and their relationship with cognitive function.

**Methods:** Thirty T2DM patients and 20 healthy controls (HCs) were recruited and underwent 3-dimensional, high-resolution T1-weighted sequence (3D-T1) and a battery of cognitive tests. Freesurfer 6.0 was performed to segment the hippocampus into 12 subregions automatically. Then relationships between hippocampal subfield volumes and neurocognitive scale scores in the T2DM group were evaluated.

**Results:** Immediate memory scores on the auditory verbal learning test (AVLT) and Montreal Cognitive Assessment (MoCA) scores in T2DM patients were lower than in the HCs. T2DM patients showed that volumes of the bilateral hippocampus were significantly reduced, mainly in the bilateral molecular layer, granule cell and molecular layer of the dentate gyrus (GC-ML-DG), cornu ammonis 4 (CA4), fimbria, and left subiculum and the right hippocampus amygdala transition area (HATA) compared to HCs. In addition, T2DM patients showed the FINS was negatively correlated with volume of left GC-ML-DG (*r* = −0.415, *P* = 0.035) and left CA4 (*r* = −0.489, *P* = 0.011); the FBG was negatively correlated with volume of right fimbria (*r* = −0.460, *P* = 0.018); the HOMA-IR was negatively correlated with volume of left GC-ML-DG (*r* = −0.367, *P* = 0.046) and left CA4(*r* = 0.462, *P* = 0.010). Partial correlation analysis found that the volume of right HATA in T2DM group was positively correlated with AVLT (immediate) scores (*r* = 0.427, *P* = 0.03).

**Conclusion:** This study showed the volumes of multiple hippocampal subfields decreased and they were correlated with FINS, FBG and HOMA-IR in T2DM patients. We hypothesized that decreased hippocampal subfields volumes in T2DM patients was related to insulin resistance and impaired vascular function. In addition, we also found that abnormal hippocampal subfields volumes were related to memory function in T2DM patients, suggesting that reduced volumes in specific hippocampal subfields may be the potential mechanism of memory dysfunction in these patients.

## Introduction

Cognitive impairment is a costly medical problem worldwide and it is likely to develop into dementia. Type 2 diabetes mellitus (T2DM) is one of the major factors giving rise to the global incidence of cognitive impairment (1, 2).

Previous studies have shown that various metabolic abnormalities caused by T2DM were closely related to the cognitive decline, and insulin resistance (IR) seemed to be one of the more important factors (3, 4). Insulin receptors are widely expressed in neurons and glial cells throughout the brain (5, 6). Insulin from the pancreas binds to insulin receptors in the brain, initiating two different signaling cascades. The phosphoinositide 3-kinase path controls metabolism. The mitogen activated protein kinase path regulates mitochondrial function, proliferation and growth. Changes in these signaling cascades impede insulin sensitivity and lead to insulin resistance in the brain, thereby affecting neuronal structural plasticity and cognitive status (7). The hippocampus may be vulnerable to the effects of cerebral insulin resistance, because there is an abundant expression of insulin receptors in the hippocampus (8). In the meantime, the hippocampus is a significant region which is one of the major components of the limbic system that regulates the memory function, sensation and emotion of the brain. Cognitive impairment in T2DM patients is closely related to changes in hippocampal structure and function (9–11). Animal models have shown that IR in the hippocampus is a potential mediator of cognitive dysfunction in T2DM (12).

The hippocampus is considered to be a highly complex and heterogeneous structure. It is not structured consistently due to its different subregions perform their respective functions. The subregions of the hippocampus differ anatomically and functionally (13). These subfields constitute the internal loop of the hippocampus and coordinate the functions of the hippocampus (14). Recent studies had shown that different subregions of the hippocampus differ in their sensitivity to aging, neurological and psychiatric diseases due to uneven loss of neurons (13). Results from some related studies indicated that hippocampal atrophy was related to cognitive impairment in a variety of diseases (15, 16). To date, the hippocampus has been treated as a whole structure in most studies examining hippocampal injury (17–19), but few research has considered the subdivisions of hippocampal atrophy in T2DM patients. The relationship between the structural changes in these hippocampal subfields and cognitive decline, as well as cognitive impairment in patients with T2DM, remains unclear.

In this study, we used FreeSurfer 6.0 software to explore morphological changes of the hippocampus and its subfields in T2DM patients and their relationship with cognitive function.

## Methods

### Participants

Participants were included from the First Affiliated Hospital of Guangzhou University of Chinese Medicine from January 2018 to July 2019. This study was approved by the Medical Research Ethics Committee of Guangzhou University of Chinese Medicine, and written informed consent was provided by all subjects.

Diagnosis were conducted by an endocrinologist using standard criteria according to American Diabetes Association (20). The healthy controls (HCs) were healthy people who have had physical examinations. The participants were Han Chinese and native Chinese speakers, and all of them were right-handed. The exclusion criteria were as follows: (1) age <18 or >65 years old; (2) organic central nervous system disease; (3) history of mental and psychological disease and family history; (4) history of severe head trauma; (5) severe hypoglycemic history; (6) micro- and macrovascular complications; (7) history of alcohol dependence and poison use; (8) obvious hearing or visual impairments; (9) pregnancy, breastfeeding, and current contraceptive use (applicable to women); and (10) contraindications for magnetic resonance imaging (MRI) examination. General demographic data of all the participants was obtained by self-report, including sex, age, and education. In total, 30 T2DM patients and 20 HCs were included in this study.

### Clinical Measurements

Clinical biochemical measurements of T2DM patients included HbA1c, fasting blood glucose (FBG), fasting insulin (FINS). HOMA-IR was calculated by HOMA-IR = FBG × FINS/22.5.

### Cognitive Testing

All participants underwent a comprehensive series of neuropsychological tests, including the Montreal Cognitive Assessment (MoCA) (21), auditory verbal learning test (AVLT, include immediate recall, 5-min delayed recall, 20-min delayed recall, and recognition) (22) and grooved pegboard test (GPT) (23). These tests took ~30 min to finish.

### MRI Acquisition

MRI data was acquired on a 3-T GE SIGNA clinical MRI scanner with an eight-channel phased-array head coil. Scanning consisted of two parts: conventional brain axial T1-weighted and fluid-attenuated inversion recovery (FLAIR) images were used to rule out brain organic diseases, and a 3-dimensional, high-resolution sagittal T1-weighted (3D-T1) sequence scan was used for experimental processing. The details of the data acquisition were consistent with our previous studies (24).

### Small-Vessel Disease Assessment

Quantitative assessment of WMH and lacunar infarcts were performed on FLAIR images with ARWMC Wahlund scoring rules (25) of five regions, including the bilateral frontal lobes, parietal and occipital lobes, temporal lobes, cerebellum and brain stem, and basal ganglia. All participants with a rating score > 2 were excluded. Two experienced raters blinded to group allocations per-formed the ratings independently.

### Data Processing

FreeSurfer is brain reconstruction software that can directly and automatically segment deep subcortical gray matter structures based on voxel signals and can provide a wide range of automated neuroimaging analyses (26). FreeSurfer which can accurately segment the hippocampus (27–30), is more efficient and effective than manual segmentation (31, 32), and has been widely used in the study of hippocampal subfields. T1-weighted images were processed by the image-processing pipeline from FreeSurfer 6.0 (http://surfer.nmr.mgh.harvard.edu/), and then the hippocampal subfields were automatically segmented to obtain the volumes of hippocampal subfields and estimated the total intracranial volume.

Please refer to descriptions in the previous literature (33, 34) for the specific processing technology details of FreeSurfer. Finally, two experienced radiologists confirmed the accuracy of the segmentation of the deep subcortical gray matter structure of each participant and excluded those with neurological diseases. To reduce the influence of individual variation, we used estimated intracranial volume (eTIV) as a covariate.

**Figure 1:**
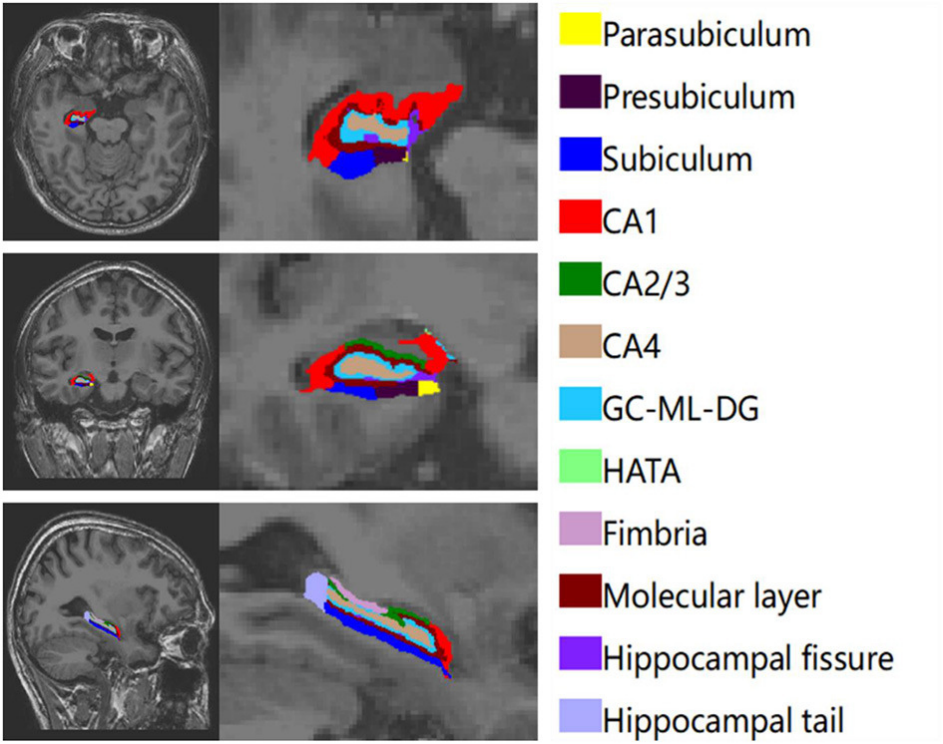
A simple of left hippocampal subfield automated segmentation.

The hippocampus was divided into 12 subregions. We obtained the entire volumes of the bilateral hippocampus, the volume of each subfield and eTIV. Figure 1 shows the automatic segmentation of one patients' hippocampus in our study.

### Statistics

Statistical Package for the Social Sciences (IBM, SPSS, version 26) was used to conduct statistical analysis. For continuous variables, it was expressed as means and standard deviations by using independent two-sample *t*-tests if it met normal distribution. If not, it was showed by using Mann-Whitney test. x^2^ test was used for proportions. The significance level was set at *P* < 0.05.

Group comparisons in hippocampal subfields volumes were run using covariance analysis (ANCOVA) with controlled age, sex, education, and eTIV. For the processed image data of hippocampal subfields, we used false discovery rate (FDR) method for correction. The significance level was set at a Benjamini and Hochberg (B-H) correction-adjusted *P* value (*q*) < 0.05.

Partial correlation analysis was performed to evaluate the correlation of significantly reduced hippocampal subfields volumes with AVLT (immediate) and clinical biochemical parameters (HbA1c, FINS, FBG) in T2DM patients, with gender, age, education level, and eTIV as covariables. Then partial correlation analysis was again used to evaluate the correlation between AVLT (immediate) and clinical biochemical parameters (HbA1c, FINS, FBG) in T2DM patients, with gender, age and education level as covariables. *P* < 0.05 was considered statistically significant.

Because MoCA and HOMA-IR do not meet normal distribution. We applied linear regression equation to obtain the residual between AVLT (immediate) and MoCA score after regression of sex, age and education level, and the residual of hippocampal subfields volumes after regression of sex, age, education level and eTIV. Spearman correlation analysis was used to investigate the correlation between HOMA-IR and residuals of AVLT (immediate) and MoCA, and the correlation between HOMA-IR and residuals of hippocampal subfields volumes. The correlations between MoCA and HbA1c, FINS, FBG and hippocampal subfields volumes were also calculated. *P* < 0.05 was considered statistically significant.

## Results

### Demographic Results

There were no significant differences in age, sex, or education levels between the two groups. The demographic information and clinical biochemical index information is shown in Table 1.

**Table 1:** Demographic data and clinical biochemical indicators of all subjects.

	**HC** **(***n*** = 20)**	**T2DM** **(***n*** = 30)**	**Statistics**	***P*** **value**
Age (years)	49.20 ± 5.21	50.93 ± 8.93	*t* = −0.865	0.391
Sex (male, %)	11 (55)	17 (56.7)	*χ^2^* = 0.014	0.907
Education (years)	9 (7.5, 12)	9 (9, 12)	*z* = −0.664	0.507
eTIV (mm^3^)	1491495.39 ± 129183.82	1444202.96 ± 105401.43	*t* = 1.420	0.162
HbA1c (%, x ± s)	N/A	9.30 ± 2.04	N/A	N/A
FINS (μIU/ml, x ± s)	N/A	6.89 ± 2.52	N/A	N/A
FBG (mmol/L, x ± s)	N/A	8.16 ± 2.61	N/A	N/A
HOMA-IR	N/A	2.02 (1.68, 3.04)	N/A	N/A

*eTIV, estimated total intracranial volume; FBG, fasting blood glucose; FINS, fasting insulin; N/A, Not applicable*.

### Cognitive Assessments

The T2DM group had worse performance on the AVLT (immediate) and MoCA tests than the HC group. However, there were no significantly differences in AVLT (5 min), AVLT (20 min), AVLT (recognition), GPT (R), and GPT (L) scores between the two groups. All raw scores for the cognitive tests are reported in Table 2.

**Table 2:** Neuropsychological result of all group.

	**HC** **(***n*** = 20)**	**T2DM** **(***n*** = 30)**	**Statistics**	***P*** **value**
AVLT (immediate)	23.10 ± 4.71	19.80 ± 4.87	*t* = 2.379	0.021*
AVLT (5 min)	7.5 (7, 10)	8 (5.75, 9)	*z* = −0.450	0.652
AVLT (20 min)	8 (7, 9)	8 (5.75, 9)	*z* = −0.511	0.610
AVLT (recognition)	12 (10, 12)	11.5 (9.75, 12)	*z* = −0.622	0.534
MoCA score	27 (26, 29)	26 (22, 27)	*z* = −2.843	0.004*
GPT (R) (s)	80.00 (67.50, 86.43)	82.40 (70.60, 97.62)	*z* = −1.377	0.168
GPT (L) (s)	80.10 (76.00, 98.50)	86.90 (77.50, 99.95)	*z* = −0.872	0.383

*MoCA, montreal cognitive assessment; AVLT, auditory verbal learning test; GPT, grooved pegboard test*.

* *P < 0.05*.

### Hippocampal Subfields Analysis

**Figure 2:**
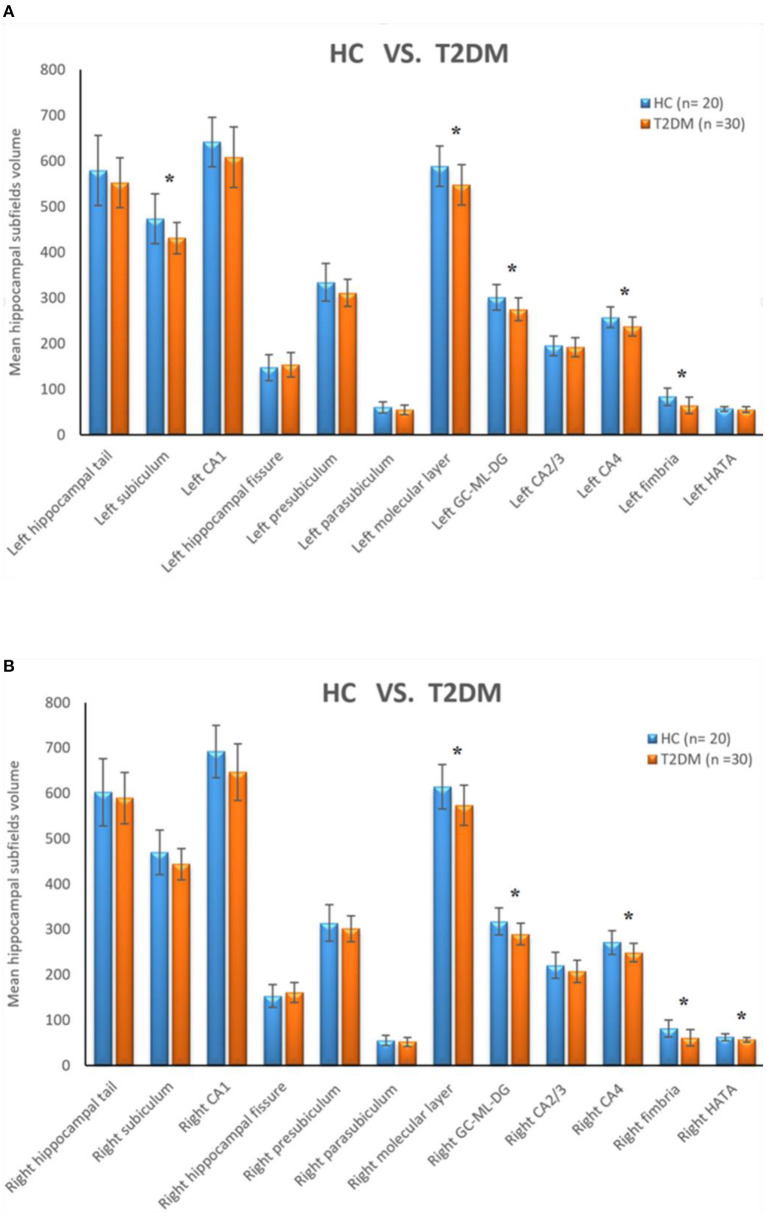
**(A)** Comparison of the volumes of left hippocampal subfields between HC group and T2DM group. **(B)** Comparison of the volumes of right hippocampal subfields between HC group and T2DM group. *Correction adjusted *P* value (*q*) < 0.05.

The bilateral hippocampus was divided into 24 subfields, including the bilateral parasubiculum, presubiculum, subiculum, cornu ammonis 1-4 (CA1-4), granule cell and molecular layer of the dentate gyrus (GC-ML-DG), hippocampus amygdala transition area (HATA), fimbria, molecular layer, hippocampal fissure and hippocampal tail. Among them, CA2/3 belongs to one region. The volumes of the total hippocampus and its subfields are shown in Table 3; Figure 2. We found that volumes of the bilateral hippocampus in T2DM group were significantly reduced, mainly in the bilateral molecular layer, GC-ML-DG, CA4, fimbria, and left subiculum and the right HATA. No significant differences were found in the volumes of the remaining hippocampal subfields between the two groups.

**Table 3:** Group comparison of hippocampal subfields volume.

	**HC** **(***n*** = 20)**	**T2DM** **(***n*** = 30)**	***F*** **value**	***P*** **value**	***q*** **value**	***partial eta*** **^2^**
Left hippocampal tail	579.05 ± 77.01	552.49 ± 54.49	1.757	0.192	0.263	0.038
Left subiculum	473.15 ± 54.25	430.64 ± 33.78	10.473	0.002	0.017*	0.192
Left CA1	641.28 ± 53.76	607.83 ± 65.95	2.182	0.147	0.225	0.047
Left hippocampal fissure	146.95 ± 28.34	153.76 ± 26.71	1.45	0.235	0.306	0.032
Left presubiculum	334.09 ± 41.40	310.76 ± 30.03	4.374	0.042	0.084	0.090
Left parasubiculum	59.89 ± 12.20	54.35 ± 10.50	3.256	0.078	0.127	0.069
Left molecular layer	588.10 ± 44.03	547.13 ± 44.22	8.146	0.007	0.020*	0.156
Left GC-ML-DG	300.92 ± 27.88	274.62 ± 24.96	8.877	0.005	0.019*	0.168
Left CA2/3	194.59 ± 21.62	191.19 ± 21.02	0.092	0.763	0.763	0.002
Left CA4	257.06 ± 22.77	236.76 ± 20.68	6.922	0.012	0.028*	0.136
Left fimbria	83.20 ± 19.23	63.92 ± 18.05	11.608	0.001	0.013*	0.209
Left HATA	56.37 ± 5.25	54.76 ± 6.51	0.113	0.739	0.769	0.003
Left whole hippocampus	3567.70 ± 266.34	3324.40 ± 239.99	9.461	0.004	0.017*	0.177
Right hippocampal tail	602.24 ± 74.05	589.51 ± 56.89	0.168	0.684	0.741	0.004
Right subiculum	469.78 ± 49.06	443.72 ± 34.80	3.395	0.072	0.125	0.072
Right CA1	691.99 ± 57.26	646.84 ± 62.27	4.097	0.049	0.091	0.085
Right hippocampal fissure	153.39 ± 24.97	161.29 ± 22.34	1.96	0.168	0.243	0.043
Right presubiculum	313.72 ± 40.19	301.59 ± 28.59	0.751	0.391	0.462	0.017
Right parasubiculum	55.45 ± 11.18	52.08 ± 9.71	0.616	0.437	0.494	0.014
Right molecular layer	614.52 ± 48.97	573.55 ± 44.29	6.682	0.013	0.028*	0.132
Right GC-ML-DG	317.47 ± 29.96	289.77 ± 24.32	9.856	0.003	0.020*	0.183
Right CA2/3	220.62 ± 28.39	207.70 ± 24.38	1.226	0.274	0.339	0.027
Right CA4	271.65 ± 26.29	248.70 ± 20.16	8.596	0.005	0.016*	0.163
Right fimbria	81.72 ± 18.89	60.93 ± 17.93	18.993	<0.001	<0.026*	0.302
Right HATA	62.69 ± 7.50	56.77 ± 5.34	9.852	0.003	0.016*	0.183
Right whole hippocampus	3701.86 ± 286.18	3471.11 ± 234.58	7.553	0.009	0.023*	0.147

*Adjusted age, sex, education, and eTIV. CA, cornu ammonis; GC, granular cell; ML, molecular layer; DG, dentate gyrus; HATA, hippocampus amygdala transition area. Correction adjusted P value (q value) < 0.05 had statistical significance*.

* *Correction adjusted P value (q value) < 0.05*.

### Correlation Analysis of Hippocampal Subfields Volumes and Clinical Biochemical Index

**Figure 3:**
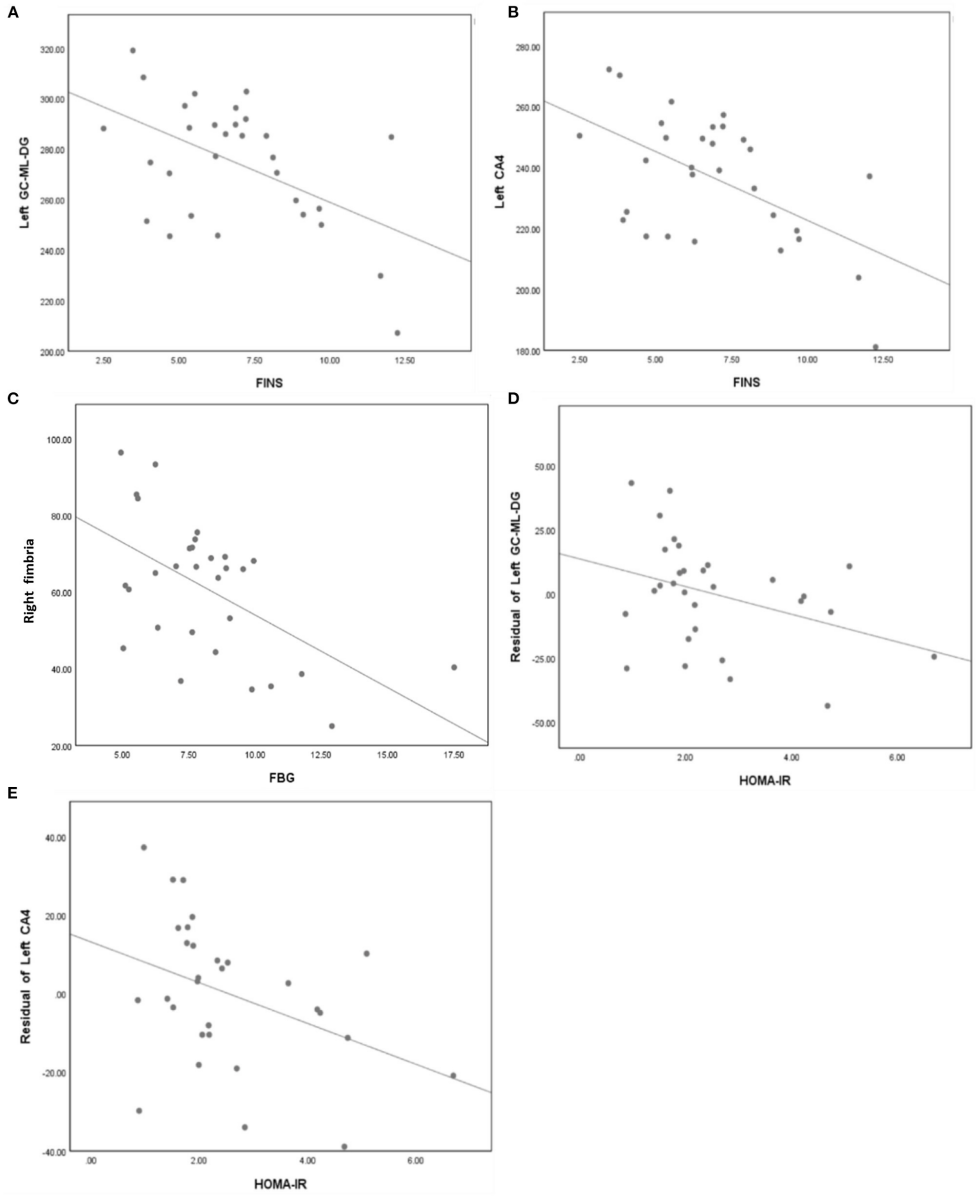
**(A)** FINS was negatively correlated with volume of left GC-ML-DG (*r* = −0.415, *p* = 0.035). **(B)** FINS was negatively correlated with volume of left CA4 (*r* = −0.489, *p* = 0.011). **(C)** FBG was negatively correlated with volume of right fimbria (*r* = −0.460, *p* = 0.018). **(D)** HOMA-IR was negatively correlated with volume of left GC-ML-DG (*r* = −0.367, *p* = 0.046). **(E)** HOMA-IR was negatively correlated with volume of left CA4 (*r* = −0.462, *p* = 0.010).

In the T2DM group, partial correlation analysis found that the FINS was negatively correlated with volume of left GC-ML-DG (*r* = −0.415, *P* = 0.035) and left CA4 (*r* = −0.489, *P* = 0.011); the FBG was negatively correlated with volume of right fimbria (*r* = −0.460, *P* = 0.018). And spearman correlation analysis found that the HOMA-IR was negatively correlated with volume of left GC-ML-DG (*r* = −0.367, *P* = 0.046) and left CA4 (*r* = −0.462, *P* = 0.010) (Figure 3).

### Correlation Analysis of Cognitive Function and Clinical Biochemical Index

There was no correlation between cognitive scores [AVLT (immediate) and MoCA] and clinical biochemical indexes (HbA1c, FBG, FINS, HOMA-IR) in T2DM group.

### Correlation Analysis of Hippocampal Subfield Volumes and Cognitive Function

**Figure 4:**
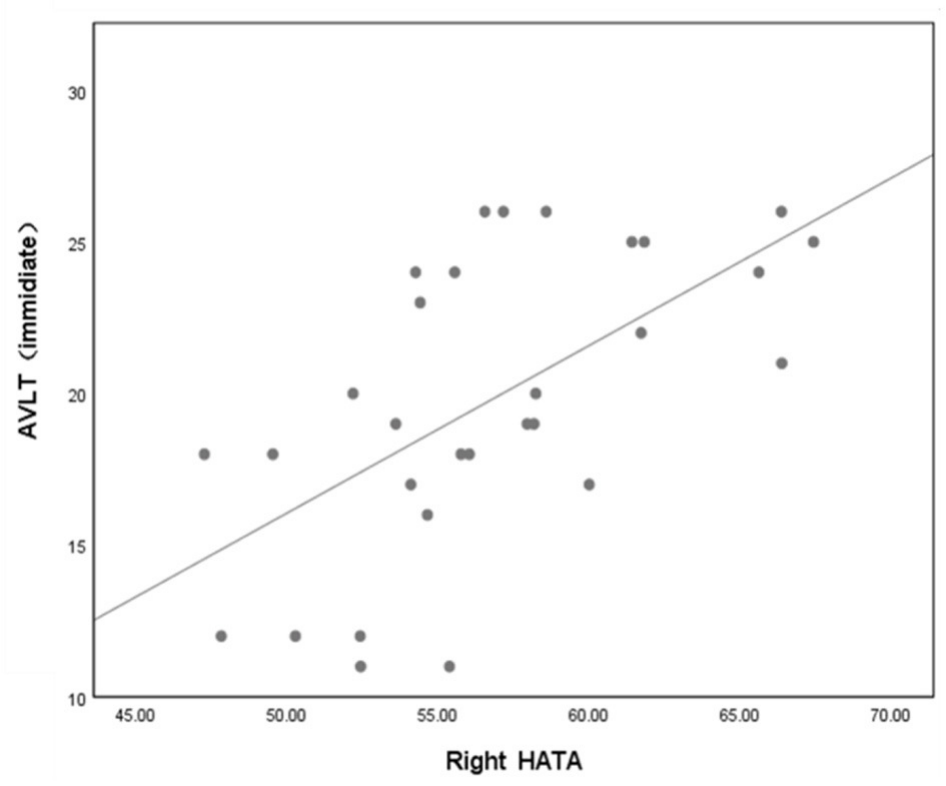
Volume of right HATA was correlated with AVLT (immediate) scores (*P* = 0.03, *r* = 0.427). Adjusted age, sex, education and eTIV. AVLT, auditory verbal learning test; HATA, hippocampus amygdala transition area.

In the T2DM group, partial correlation analysis found that the volume of right HATA was positively correlated with AVLT (immediate) scores (*r* = 0.427, *P* = 0.03) (Figure 4).

## Discussion

The MoCA assesses the cognitive ability of subjects from the perspective of overall cognitive function and is currently widely used in cognitive-related research. In our study, we found that T2DM patients has lower MoCA scores than HCs, which was consistent with our previous studies (35). In addition, we found that the AVLT (immediate) scores were lower in the T2DM patients, suggesting that T2DM patients had immediate memory impaired. It had previously been shown that T2DM patients have impaired cognitive function, which mainly manifested as a slowdown in information processing, memory and attention loss, and disruptions in executive function and visual spatial abilities (36, 37). In this study, performance on the GPT was worse in the T2DM group than in the HCs, but the difference was not statistically significant that might be due to the small sample size that failed to arrive at a significant difference.

In previous studies, abnormalities of the hippocampus in terms of structure and function have been shown to be related to T2DM (17, 38). In this study, we also found that the bilateral total hippocampal volumes in T2DM patients were reduced. However, few research has considered the subfields of hippocampal reduced in T2DM patients. This study further evaluated hippocampal subfield volumes in T2DM patients. Compared with the HCs, the T2DM patients showed decreased volumes of hippocampal subfields in the bilateral molecular layers, GC-ML-DG, CA4, fimbria, left subiculum and right HATA. In addition, we also found that in T2DM patients the FINS was negatively correlated with volume of left GC-ML-DG and left CA4; the FBG was negatively correlated with volume of right fimbria; HOMA-IR was negatively correlated with volume of left GC-ML-DG and left CA4. Our results suggested that hyperglycemia and IR in T2DM patients were closely related to decreased hippocampal subfields volumes. Insulin and its receptors are widely expressed in the brain and play a critical role in neuronal proliferation and differentiation (39, 40). The subgranular zone, which is located in the dentate gyrus of the hippocampus, is one of the two major neural stem cell regions of the adult brain. Insulin and insulin-like growth factors (IGFs) play an important role in neural stem cell self-renewal and neurogenesis through different ligand-receptor interactions (41). Insulin has previously been reported to promote dendritic spines formation in rat hippocampal neurons. Conversely, the use of blocking antibodies or down-regulation of IR signal resulted in a decrease in dendritic spines (42). In addition, studies have shown that entorhinal cortex stimulation promotes neurogenesis in the hippocampal DG region of adult rats, while insulin receptor antagonists attenuated neurogenesis (43). Therefore, we hypothesized that insulin resistance might weaken neurogenesis in the dentate gyrus of the hippocampus, leading to the decrease in the volumes of the hippocampal subfields. In addition, hyperinsulinemia/insulin resistance is associated with the promotion of atherosclerosis (44). Hyperglycemia is one of the main causes of vascular dysfunction and injury in T2DM patients, but the mechanism of its harmful effects is still unclear. According to previous studies, it mainly involves the following four theories (a) Aldose-reductase, polyol pathway; (b) non-enzymatic glycation; (c) alteration of redox potential; and (d) diacylgly-cerol-protein kinase C pathway (45). Therefore, we speculated that vascular dysfunction and injury might be another reason for the decrease of hippocampal subfields volumes. Unfortunately, this study did not observe the cerebrovascular changes in T2DM patients, and we need to further study the relationship between cerebrovascular changes and decreased hippocampal subfields volumes in T2DM patients in the future.

Correlation analysis between the atrophic hippocampal subfields volumes and different neurocognitive scale scores showed that R-HATA's volume was positively correlated with AVLT (immediate) scores, suggesting that the damage to the HATA was closely related to patient memory loss, which was partly similar to the results of Zheng's research. According to previous studies, atrophy of the HATA may damage the integrity of the hippocampus-amygdala network, affect information processing, and promote cognitive dysfunction (16). In addition, the subiculum was connected to the deep part of the entorhinal cortex and other cortical and subcortical parts and was the outflow pathway for hippocampal signals. In hippocampal-dependent memory tasks, the subiculum was shown to be involved in memory retrieval, and the entorhinal cortex was shown to be involved in temporary retention (13). Previous studies have also found that structural abnormalities in the hippocampal CA1 region and subiculum in patients with T2DM were closely related to MoCA scores and delayed memory scores (46). Unfortunately, although this study found atrophy of the left subiculum in patients with T2DM, it failed to verify the correlation between the size of the subiculum and cognitive function. Perhaps the sample size needs to be increased for further research in the future.

### Limitations

First, the sample size in this study was small. Second, we had comprehensive indicators related to diabetes, while lacking of disease duration records, which will be included in future studies. Finally, this was only a single modal study examining the structure of the hippocampus, which failed to fully consider the relationship between the hippocampus and other brain regions in the whole brain. Multiparametric techniques should be applied in future studies.

## Conclusions

This study showed the volumes of multiple hippocampal subfields decreased and they were correlated with FINS, FBG and HOMA-IR in T2DM patients. We hypothesized that decreased hippocampal subfields volumes in T2DM patients was related to insulin resistance and impaired vascular function. In addition, we also found that abnormal hippocampal subfields volumes were related to memory function in T2DM patients, but how the structural changes of hippocampal subfields affect the cognitive state of T2DM patients still needs to be further explored.

## Data Availability Statement

The raw data supporting the conclusions of this article will be made available by the authors, without undue reservation.

## Ethics Statement

The studies involving human participants were reviewed and approved by Medical Research Ethics Committee of Guangzhou University of Chinese Medicine. The patients/participants provided their written informed consent to participate in this study.

## Author Contributions

MRL and YFL designed the whole experiments and finished the manuscript. YJL contributed to the statistical analysis. HMH and XL administered the neuropsychological tests. YNC, YF, and XMM collected data of the subjects. YL and XT did the data analysis and amended the manuscript. SJQ was the guarantor of this study and chiefly responsible for the whole process of the experiment. All authors contributed to the article and approved the submitted version.

## Funding

This study was supported by the Key International Cooperation Project of National Natural Science Foundation of China (81920108019) and the Medical Scientific Research Foundation of Guangdong Province (A2021182). YL was also supported by Excellent Doctoral Dissertation Incubation Grant of First Clinical School of Guangzhou University of Chinese Medicine (YB202003).

## Conflict of Interest

The authors declare that the research was conducted in the absence of any commercial or financial relationships that could be construed as a potential conflict of interest.

## Publisher's Note

All claims expressed in this article are solely those of the authors and do not necessarily represent those of their affiliated organizations, or those of the publisher, the editors and the reviewers. Any product that may be evaluated in this article, or claim that may be made by its manufacturer, is not guaranteed or endorsed by the publisher.
